# Exploring cognitive and biological correlates of sleep quality and their potential links with Alzheimer’s disease (ALFASleep project): protocol for an observational study

**DOI:** 10.1136/bmjopen-2022-067159

**Published:** 2022-12-30

**Authors:** Karine Fauria, Carolina Minguillon, Iva Knezevic, Núria Tort-Colet, Laura Stankeviciute, Laura Hernández, Andreea Rădoi, Carme Deulofeu, Sherezade Fuentes-Julián, Israel Turull, David Fusté, Gonzalo Sánchez-Benavides, Eider M Arenaza-Urquijo, Marc Suárez-Calvet, Sebastian C Holst, Pilar Garcés, Thomas Mueggler, Henrik Zetterberg, Kaj Blennow, Aurora Arqueros, Álex Iranzo, Juan Domingo Gispert, José Luis Molinuevo, Oriol Grau-Rivera

**Affiliations:** 1 Barcelonaβeta Brain Research Center, Barcelona, Spain; 2 Centro de Investigación Biomédica en Red de Fragilidad y Envejecimiento Saludable, Instituto de Salud Carlos III, Madrid, Spain; 3 IMIM (Hospital del Mar Medical Research Institute), Barcelona, Spain; 4 Pasqual Maragall Foundation, Barcelona, Spain; 5 Servei de Neurologia, Hospital del Mar, Barcelona, Spain; 6 F Hoffmann-La Roche, Basel, Switzerland; 7 Department of Psychiatry and Neurochemistry, Institute of Neuroscience and Physiology, Sahlgrenska Academy, University of Gothenburg, Gothenburg, Sweden; 8 Clinical Neurochemistry Laboratory, Sahlgrenska University Hospital, Mölndal, Sweden; 9 UK Dementia Research Institute at UCL, London, UK; 10 Department of Neurodegenerative Disease, UCL Queen Square Institute of Neurology, London, UK; 11 Hong Kong Center for Neurodegenerative Diseases, Hong Kong, People's Republic of China; 12 Neurology Service, Hospital Clínic de Barcelona and Institut D'Investigacions Biomèdiques August Pi i Sunyer (IDIBAPS), Barcelona, Spain; 13 Centro de Investigación Biomédica en Red sobre Enfermedades Neurodegenerativas (CIBERNED), Instituto de Salud Carlos III, Madrid, Spain; 14 Centro de Investigación Biomédica en Red de Bioingeniería, Biomateriales y Nanomedicina (CIBER-BBN), Instituto de Salud Carlos III, Madrid, Spain

**Keywords:** Dementia, SLEEP MEDICINE, NEUROLOGY

## Abstract

**Introduction:**

The growing worldwide prevalence of Alzheimer’s disease (AD) and the lack of effective treatments pose a dire medical challenge. Sleep disruption is also prevalent in the ageing population and is increasingly recognised as a risk factor and an early sign of AD. The ALFASleep project aims to characterise sleep with subjective and objective measurements in cognitively unimpaired middle/late middle-aged adults at increased risk of AD who are phenotyped with fluid and neuroimaging AD biomarkers. This will contribute to a better understanding of the pathophysiological mechanisms linking sleep with AD, thereby paving the way for the development of non-invasive biomarkers and preventive strategies targeting sleep.

**Methods and analysis:**

We will invite 200 participants enrolled in the ALFA+ (for ALzheimer and FAmilies) prospective observational study to join the ALFASleep study. ALFA+ participants are cognitively unimpaired middle-aged/late middle-aged adults who are followed up every 3 years with a comprehensive set of evaluations including neuropsychological tests, blood and cerebrospinal fluid (CSF) sampling, and MRI and positron emission tomography acquisition. ALFASleep participants will be additionally characterised with actigraphy and CSF–orexin-A measurements, and a subset (n=90) will undergo overnight polysomnography. We will test associations of sleep measurements and CSF–orexin-A with fluid biomarkers of AD and glial activation, neuroimaging outcomes and cognitive performance. In case we found any associations, we will test whether changes in AD and/or glial activation markers mediate the association between sleep and neuroimaging or cognitive outcomes and whether sleep mediates associations between CSF–orexin-A and AD biomarkers.

**Ethics and dissemination:**

The ALFASleep study protocol has been approved by the independent Ethics Committee Parc de Salut Mar, Barcelona (2018/8207/I). All participants have signed a written informed consent before their inclusion (approved by the same ethics committee). Study findings will be presented at national and international conferences and submitted for publication in peer-reviewed journals.

**Trial registration number:**

NCT04932473.

**Strengths and limitations of this study**

- The study has a multimodal approach combining not only subjective but also objective sleep measurements, cerebrospinal fluid orexin determinations, as well as fluid and neuroimaging Alzheimer’s disease (AD) biomarkers.
- Although the study population includes cognitively unimpaired adults from a cohort that has been enriched for AD risk factors, which may limit the generalisability of the results to the general population, this recruitment strategy has been optimised to identify early AD pathophysiological events.
- An additional source of bias is that the study population mostly comprises well-educated individuals with very low comorbidity burden and low ethnic diversity.
- The design is that of a cross-sectional study nested in a prospective observational cohort, enabling a potential follow-up study with longitudinal sleep measurements.
- The observational design of the study does not allow us to infer causality in potential associations between sleep and AD-related outcomes.

## Introduction

### Alzheimer’s disease (AD)

AD is one of the leading public health problems worldwide, potentially affecting 100 million people by 2050.^1^ Currently available fluid and neuroimaging biomarkers allow the in vivo detection of brain amyloid [amyloid beta (Aβ)] and tau pathology (the AD neuropathological hallmarks), with evidence showing that these proteins start to accumulate ~10 to 20 years before the onset of cognitive impairment.^2^ This preclinical stage represents an exceptional window of opportunity for preventive interventions targeting potentially modifiable risk factors, which have been pointed to account for up to 40% of dementia risk.^3^

### Sleep and cognitive impairment

Sleep alterations have been consistently associated with worse cognitive outcomes,^4^ and sleep disturbances are common in patients with AD.^5 6^ The latter has been related to the neurodegeneration of neural systems that regulate the sleep–wake state.^7 8^ Nevertheless, research from the last decade suggests that sleep disorders may be a risk factor for cognitive impairment.^4 9–12^ In line with this, obstructive sleep apnoea (OSA) treatment may slow disease progression in patients with mild cognitive impairment (MCI).^13^ Conversely, while non-pharmacological interventions may improve sleep quality in patients with MCI, whether such interventions can prevent or delay cognitive impairment remains to be demonstrated.^14 15^ In light of this evidence, sleep is emerging as a potential candidate for strategies aimed to prevent cognitive impairment. However, to design such interventions, a better understanding of mechanisms linking sleep with cognitive impairment is needed.

### Sleep and AD: a bidirectional relationship?

Different lines of research point to a complex interplay between sleep and AD pathophysiology, with reciprocal effects on each other.^16^ First, evidence from animal models^17^ and human studies^18 19^ suggests that sleep deprivation promotes Aβ peptide and tau protein accumulation in the brain due to increased neuronal activity and impaired function of the glymphatic system (a physiological brain waste clearance mechanism that is strongly enhanced during sleep).^20^ Second, AD-related neurodegeneration in brain regions involved in sleep–wake cycle regulation may alter sleep and circadian regulation early in the disease.^21 22^ A potential effect of chronic sleep deprivation on AD pathology is further supported by several studies showing cross-sectional associations between altered sleep quality, sleep duration and OSA with higher Aβ pathology burden in cognitively unimpaired adults.^23–27^ Other studies have evaluated the relationship between altered sleep and tau biomarkers, with less consistent results.^27–32^ Additionally, reduced sleep efficiency and non-rapid eye movement (NREM) slow-wave activity (SWA) have been shown to predict Aβ accumulation over time.^33^ However, recent data suggest that associations between cognitive decline and these changes in sleep architecture may not be linear.^34^ Other longitudinal studies have related OSA and excessive daytime sleepiness with increased Aβ and tau burden.^35–37^ In summary, while there is evidence that sleep disturbance may act both as a risk factor and clinical manifestation of AD, mechanisms underlying these associations remain unclear, and longitudinal studies involving individuals at the very early stage of preclinical AD are needed to unravel the directionality of this relationship.

### Role of sleep oscillations

During NREM sleep, cortical electroencephalographic activity oscillates between periods of activity and silence at very low frequencies.^38^ This SWA can be further classified as slow oscillations (<1 Hz) and delta activity (1–4 Hz). Another cortical rhythm associated with NREM sleep is spindles (11–16 Hz), which originate in the reticular nucleus of the thalamus and propagate through the thalamocortical loop.^39^ Both SWA and spindle activities are reduced in normal ageing, particularly in frontal areas.^40^ However, in the context of AD pathology, these reductions are frequency-dependent and topography-dependent. In healthy older adults with increased amyloid burden, a specific reduction of the slow oscillation frequency, concomitant with an increase in the delta frequency, has been observed in frontal areas.^41^ Furthermore, reduction in spindle activities in the context of AD pathology shows specificity for fast frequencies (13–16 Hz) and parietal areas.^42^ Finally, a disruption of the coupling between spindles and slow oscillations has been associated both with normal ageing and increased tau burden.^43 44^ Based on this assumption, NREM SWA disruption and impaired slow oscillation–spindle coupling have been postulated as potential biomarkers of Aβ and tau deposition.^44^ Finally, alterations in REM-sleep duration, latency and synchrony have been reported in AD probably due to degeneration of basal forebrain cholinergic nuclei.^45 46^ However, the potential diagnostic usefulness of REM and NREM-sleep alterations in preclinical AD remains to be elucidated.

### Sleep, orexin and neuroinflammation

Orexin is a neuropeptide synthesised in the lateral hypothalamus that promotes sleep and inhibits rapid eye movement (REM) sleep. Increased orexin cerebrospinal fluid (CSF) levels have been described in MCI and AD dementia.^47 48^ Conversely, a postmortem study found 40% fewer orexinergic neurons in the hypothalamus of patients with AD compared with controls.^49^ Given this evidence, it has been hypothesised that orexin may be upregulated in early AD stages in response to neurodegeneration, which may in turn lead to impaired sleep–wake cycle regulation.^50^ However, other studies have found no differences between patients with AD and controls, and there is little information concerning CSF orexin levels in preclinical AD.^50^

Sleep disturbances have also been linked with systemic inflammation in humans, and sleep-deprived mice have been found to express higher levels of proinflammatory interleukins and microglial activation.^51 52^ While a dysregulated glial response is increasingly recognised as an important feature in AD pathogenesis,^53^ few studies have assessed the association between sleep and CSF markers of glial response.^26 31 32^

A better understanding of the role of orexin and neuroinflammation in the association between sleep and AD could lead to the identification of novel therapeutic targets to prevent or slow down cognitive decline.

### Structural neuroimaging in sleep disorders

MRI studies have reported inconsistent differences in grey matter volume in patients with sleep deprivation due to insomnia,^54^ mostly involving orbitofrontal, temporal, parietal and cingulate regions.^55–58^ Orbitofrontal and other frontal regions have also been consistently associated with self-reported poor sleep quality, inadequate sleep duration, sleep fragmentation and slow-wave sleep disruption.^59–61^ Insomnia symptoms and poor sleep quality have been also associated with lower volume in AD-vulnerable regions^58 62^ and loss of white matter integrity.^58 63^ Similarly, other studies have reported an overlap between brain areas affected by OSA and those affected by AD pathology, including the anterior cingulate, hippocampal, frontal, parietal and temporal lobes.^64 65^ A detailed understanding of the intersections between local sleep deficits and the topography of brain structural and AD pathology changes over time may offer a unique opportunity for staging and tracking AD preclinical changes.^46^

### Role of *APOE* and sex

*APOE-ε4* is the most important genetic risk factor for sporadic AD and has been suggested to modify the association between sleep and AD pathology.^66^ Similarly, two-thirds of all patients with AD are female,^67^ and women are at higher risk of sleep worsening with increasing age.^68^ A potential mechanism linking female sex with AD and sleep disorders is hormonal alterations since lower oestrogen levels have been associated both with increased Aβ neurotoxicity^69^ and sleep and circadian rhythm disruptions during perimenopausal and postmenopausal periods.^70^ These findings highlight the need for further research exploring the neurobiological basis of potential interactions between *APOE* and sex on sleep in preclinical AD to better understand which subgroups of patients would benefit the most from potential interventions targeting sleep quality.

### Knowledge gaps

Despite recent advances, there are still several unresolved issues concerning the relationship between sleep and cognitive impairment. To address these questions, there is a need for multimodal studies combining not only subjective but also objective sleep measurements with different outcomes related to AD risks, such as fluid and neuroimaging biomarkers and cognitive performance measurements.

The ALFASleep project aims to cover these knowledge gaps by acquiring subjective and objective sleep data, as well as measuring CSF orexin levels, in middle-aged/late middle-aged cognitively unimpaired individuals at increased risk of AD. To do so, this project takes advantage of the ongoing ALFA+ study, a prospective observational study conducted at the Barcelonaβeta Brain Research Center (BBRC) that involves the follow-up of cognitively unimpaired adults with *state-of-the-art* neuroimaging and cognitive measurements, as well as AD and glial activation biomarkers.^71^ Moreover, the prospective design of the ALFA+ study enables the longitudinal extension of the present study, thus providing critical knowledge to unravel the bidirectional relationship between sleep and AD.

### Hypotheses and aims

Our main hypotheses are (1) altered sleep patterns (including altered sleep macroarchitecture (eg, abnormal sleep duration, efficiency or fragmentation) and microarchitecture (eg, decreased SWA or spindle expression)) and OSA are associated with higher AD pathology and neuroinflammation levels (the latter after accounting for AD pathology); (2) higher CSF–orexin-A levels are associated with abnormal AD biomarkers; (3) altered sleep patterns and OSA are associated with brain structural differences involving AD vulnerable areas and worse cognitive performance; (4) association of altered sleep patterns and OSA with neuroimaging and cognitive outcomes is partially mediated by AD biomarkers and neuroinflammation; and (5) associations between CSF–orexin-A and AD biomarkers are mediated by changes in sleep architecture. To test these hypotheses, we will characterise 200 cognitively unimpaired participants from the ALFA+ study with actigraphy measurements, nasal flow monitoring devices and CSF–orexin-A levels, and a subgroup of 90 participants with overnight polysomnography (PSG). We will also explore the potential usefulness of objective sleep measurements to predict the presence underlying AD pathology.

## Methods and analysis

### Design and setting

The ALFASleep study (ClinicalTrials.gov) is an observational, cross-sectional study that is developed in the context of the ALFA+ study (NCT02485730), an observational, prospective, longitudinal study that has been optimally designed for the characterisation of early pathophysiological changes in preclinical AD.^71^

### Study population

The ALFA+ study includes 419 individuals (baseline mean age 61.1 years, range 48–73 years, 61% women), most of them kindred of patients with AD (55% *APOE-ε4* carriers), without evidence of cognitive impairment [Clinical Dementia Rating (CDR) scale=0, Mini–Mental State Examination (MMSE) score of ≥27, semantic fluency ≥12] or any medical condition that may interfere with cognitive performance (a detailed list of exclusion criteria is listed at online supplemental material). Any cases with clinical and/or psychometric evidence suggestive of cognitive impairment (presence of a significant cognitive complaint with worries accompanied by objective impairment, below 1.5 SD, ≤scaled score of 5 or ≤percentile 5 using the regular sociodemographically adjusted published norms) are discussed in an interdisciplinary committee composed of neurologists and neuropsychologists and excluded/discontinued from the study if this committee concludes that the participants meet the National Institute on Aging-Alzheimer's Association (NIA-AA) 2011 criteria for MCI or dementia diagnosis.^72 73^

10.1136/bmjopen-2022-067159.supp1 Supplementary data

The ALFA+ study involves the acquisition of variables related to sociodemographic, clinical, epidemiological, genetic and anthropometric measurements: comprehensive neuropsychological assessments, blood and CSF sampling, an extensive MRI protocol and amyloid (^18^F-Flutemetamol) positron emission tomography (PET) acquisitions every 3 years, as well as [^18^F]fluorodeoxyglucose (FDG) PET at baseline. In addition, 100 participants will have tau PET data available by the end of the ALFASleep study recruitment period. Table 1 shows the distribution of ALFA+ participants across different AD biomarker categories. We defined Aβ positivity (A+) as CSF Aβ42/40 of <0.071, and tau positivity (T+) as CSF p-tau of >24 pg/mL, measured with the exploratory NeuroToolKit immunoassays and the electrochemiluminescence immunoassay Elecsys phosphor‐tau(181P) CSF on a fully automated cobas e601 instrument (Roche Diagnostics International), respectively,^74^ and further classified participants in four AT biomarker profiles based on the NIA-AA research framework: A−T−, A+T−, A−T+ and A+T+.^75^

**Table 1:** AT classification of ALFA+ participants at baseline, based on CSF biomarkers (NIA-AA research framework criteria)

	n	%	Biomarker category
A−T−	249	62.7	Normal AD biomarkers
A+T−	104	26.2	AD pathological change
A+T+	31	7.8	AD
A−T+	13	3.3	Non-AD pathological change

A−, normal amyloid level; A+, altered amyloid level; AD, Alzheimer’s disease; ALFA, Alzheimer and Families; CSF, cerebrospinal fluid; T+, altered p-tau level; T−, normal p-tau level.

A total of 200 participants from the ALFA+ study will be invited to participate in the present study. Eligible participants have undergone the ALFA+ study baseline visit between 2016 and 2020, have available CSF and/or neuroimaging AD biomarker data and are being followed up in the ALFA+ study (see selection criteria in box 1). Participants will be selected based on these criteria and their AD biomarker status at baseline: n=100 participants with normal AD biomarkers (A−T−) and 100 within the AD *continuum* (A+T− or A+T+). All participants will be characterised with actigraphy, a nasal flow monitoring device and CSF–orexin-A determination. A subgroup (n=90) will also be invited to undergo an overnight PSG in the Sleep Centre of the Hospital Clinic of Barcelona. They will be selected based on their availability and their AD biomarker profile (~1/3 A−T−, 1/3 A+T− and 1/3 A+T+). For this substudy, we will exclude participants reporting current use of medications that may interfere with sleep architecture (eg, antidepressants or hypnotic medications). Data collection started on January 2021 and is expected to be finished in September 2023.

Box 1 **ALFASleep study inclusion and exclusion criteria**

Inclusion criteria

● Subjects who are currently participating in the ALFA+ study.

● Subjects from whom CSF and/or neuroimaging AD biomarkers have been acquired during the last 36 months prior to their enrolment in the ALFASleep study.

Exclusion criteria

● Presence of cognitive impairment.

● Presence of clinically relevant neurological disorder, psychiatric disorder or other medical conditions that may bias the interpretation of the study results according to the investigator criteria.

● Participants with OSA using CPAP

AD, Alzheimer’s disease; ALFA, Alzheimer and Families; CPAP, continuous positive airway pressure; CSF, cerebrospinal fluid; OSA, obstructive sleep apnoea.

### Recruitment and visits

**Figure 1:**
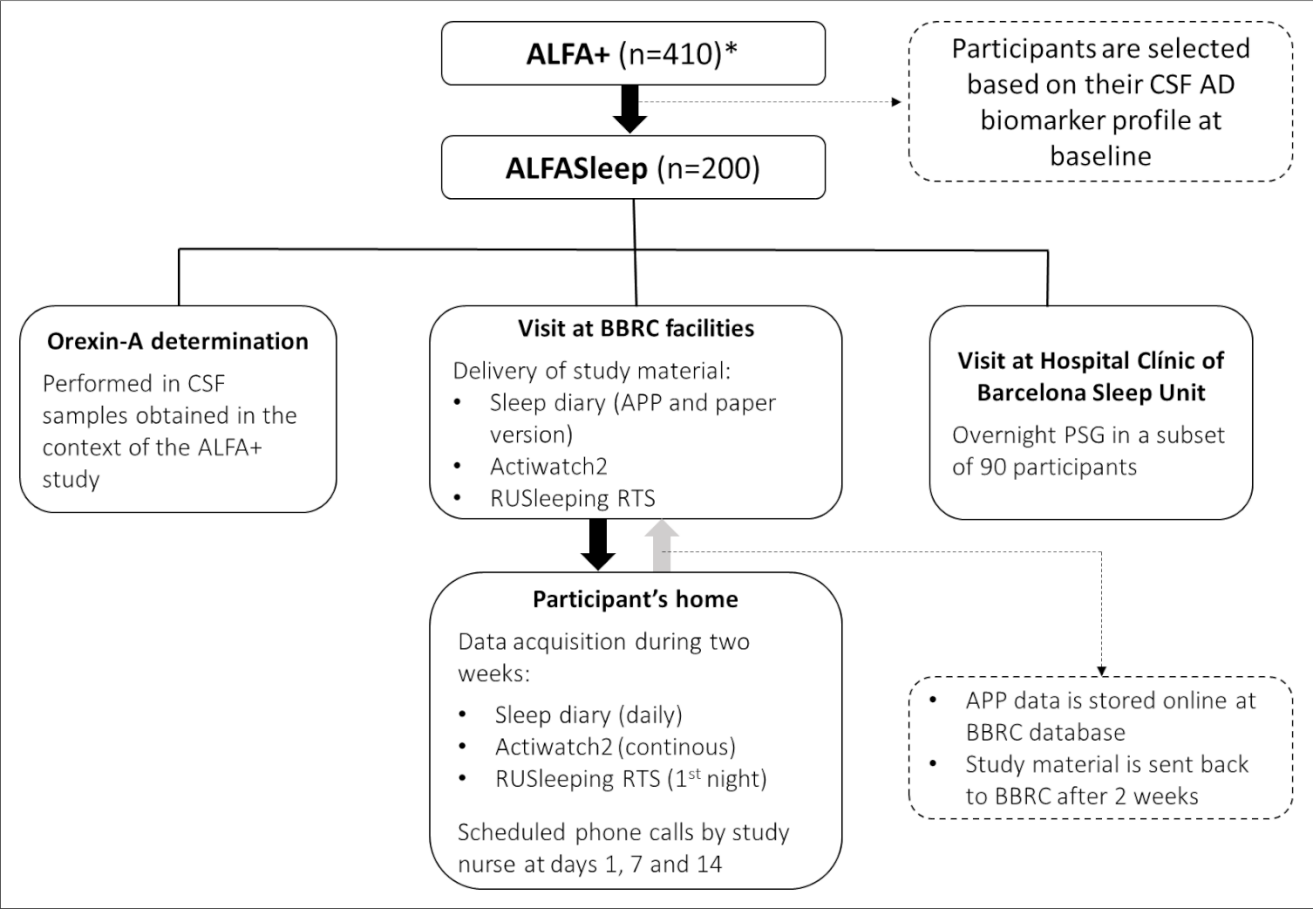
Flow diagram of the ALFASleep study. *This corresponds to the number of participants that are currently undergoing the first follow-up visit of ALFA+. AD, Alzheimer’s disease; ALFA, Alzheimer and Families; BBRC, Barcelonaβeta Brain Research Center; CSF, cerebrospinal fluid; PSG, polysomnography

We will take advantage of the nearest upcoming scheduled visit included in the ALFA+ protocol to invite 200 ALFA+ participants to join the ALFASleep study. During this session, a study nurse will explain the study and deliver a wristwatch-like actigraph (Actiwatch2; Philips Respironics, Murrysville, Pennsylvania, USA) and a nasal flow monitoring device (RUSleeping RTS, Philips Respironics) and will instal an in-house app-based electronic sleep diary in their cell phones (participants will be given a paper version of this electronic sleep diary that can be used in case they cannot use the app). Ninety participants will also be invited to the PSG substudy and will sign a separate informed consent form. A flow diagram is depicted in figure 1. Additional details about the acquisition of actigraphy, nasal flow device and PSG data are provided as online supplemental material. All relevant study variables (including clinical, neuropsychological, neuroimaging, fluid biomarkers and objective sleep data) will have been acquired within 36 months, with a maximum interval of 24 months between any objective sleep measurement and CSF sample obtention.

### Data acquisition and processing

#### Sleep objective measurements

- Actigraphy: Actigraphy data will be collected for 2 weeks with Actiwatch2 (Philips Respironics), which has shown a high correlation with PSG.^76 77^ Data will be processed with the Actiware software. The main sleep measurements will include estimations of (1) total bed time, (2) total sleep time, (3) sleep latency, (4) sleep efficiency (% of total sleep time out of total bed time), (5) wake time after sleep onset and (6) fragmentation index. We will also compute circadian parameters such as amplitude, phase and rest–activity pattern fragmentation measurements. Sampling frequency will be set at 30 s epochs, and wake threshold will be set at 20 counts.^76^ Actigraphy data will be reconciled with sleep diary data using a validated scoring algorithm to define rest intervals based on four inputs, ordered hierarchically according to the preference of use: (1) actigraph event marker, (2) participant’s diary entries, (3) actigraph light signal and (4) actigraph activity signal.^78^ We will exclude from the analyses actigraphy data collected on the same night when participants are wearing the nasal flow monitoring device to avoid the impact on sleep measurements of discomfort feeling associated with this device. Actigraphy measurements will be also acquired in the subset of participants undergoing overnight PSG to objectively document the participant’s sleep habits the week before performing the test.
- Nasal flow monitoring device: During the first night of the 2 weeks of actigraphy recording, participants will use a singlechannel portable device (RUSleeping RTS, Philips‐Respironics) that has been validated against a standard multichannel PSG for the detection of OSA.^79^ This device calculates a respiratory event index (average number of apnoeas and hypopneas per hour) and the number of respiratory events for each hour of recording. These measurements are stored locally and displayed on an LCD screen. Data will be entered into the study database by study personnel. In case data are not available, scores annotated by participants in the diary will be used (see online supplemental material). These data will be mainly used to adjust analyses involving actigraphy data by the presence of sleep apnoeas, which are a potential confounder, and to explore their independent association with cognitive neuroimaging and fluid biomarkers outcomes.
- Video-PSG: A subset of 90 participants will undergo a full night PSG study (Brain RT; OSG, Rumst, Belgium) at the Sleep Centre of the Hospital Clinic of Barcelona. PSG will include an electroencephalogram (EEG) of bilateral central, occipital and frontal regions; electrocardiography; electro-oculography; surface electromyography of the mentalis in the chin, bilateral anterior tibialis in the legs and bilateral flexor digitorum superficialis muscles in the forearms; nasal and oral airflow; thoracic and abdominal movements; continuous oxyhaemoglobin saturation; and synchronised audiovisual recording. Total sleep time, sleep efficiency, wake time after sleep onset, sleep stage amount and latencies, plus sleep-associated events will be scored in accordance with the *American Academy of Sleep Medicine Scoring Manual*.^80^ Respiratory events will be defined as apnoeas whenever airflow is interrupted for at least 10 s, and as hypopneas when the peak nasal pressure signal drops ≥30% from baseline and is associated with ≥3% oxygen desaturation from pre-event baseline and/or with arousal.^80^ To calculate the apnoea/hypopnea index, we will add up the number of apnoeas and hypopneas per hour of sleep. The periodic leg movements in sleep index will be defined as the number of leg movements per hour of sleep.^81^ For quantitative analyses, fast Fourier transform spectral analysis will be used to quantify the power of the EEG signal in different frequency bands, specifically from 0 Hz to 1 Hz for the slow oscillation band, from 1 Hz to 4 Hz for the delta band, from 11 Hz to 13 Hz for the slow spindles band, and from 13 Hz to 16 Hz for the fast spindles band.

#### Sleep subjective measurements

- Sleep diary: During the 2 weeks of actigraphy data acquisition, participants will fulfil a sleep diary (either electronic or paper version) and register any potential event that may potentially impact the quality or interpretation of the data (see online supplemental material for a full description of the data collected).
- Sleep questionnaires: We will use the Pittsburgh Sleep Quality Index, the Insomnia Severity Index and the Epworth Sleepiness Scale to measure sleep quality, insomnia symptoms and excessive daytime sleepiness, respectively.^82–84^ We will screen for the presence of REM-sleep behaviour disorder and restless legs syndrome with single-question formularies.^85 86^

#### CSF and blood samples

CSF and blood sampling and processing will be performed as described elsewhere,^71 74^ and the following measurements will be performed:

- Orexin-A determination: Orexin-A levels in CSF will be measured at the Clinical Neurochemistry Lab of the University of Gothenburg using an in-house radioimmunoassay method, as described elsewhere.^87^
- Core AD and glial markers: CSF total-tau and phosphorylated (P181)-tau will be measured with the electrochemiluminescence immunoassays Elecsys (Roche Diagnostics International). The rest of the biomarkers (Aβ42, Aβ40, neurogranin, neurofilament light, YKL-40, sTREM2, GFAP and S100) will be measured with the NeuroToolKit (Roche Diagnostics International), as previously described.^74^ All measurements will be performed at the Clinical Neurochemistry Laboratory, Sahlgrenska University Hospital, Mölndal, Sweden.
- *APOE* genotyping: *APOE* genotype determination has been performed as explained elsewhere in all ALFA+ participants.^71^

#### Neuroimaging

- MRI: Scans will be obtained with a 3 T scanner (Ingenia CX; Philips, Amsterdam, Netherlands). Images will be processed with Statistical Parametric Mapping (SPM) software for volumetric and functional analyses. Volumetric analyses will be performed through voxel-based morphometry using SPM general linear models. FreeSurfer V.6.0 software will be used to calculate the volume and cortical thickness in different regions of interest. Diffusion data will be analysed through tract-based-spatial statistics, implemented in FMRIB Software Library software. Seed-based and multivariate analyses, including an independent components analysis, will be used to evaluate data from task-free fMRI and to derive functional connectivity matrices.
- Amyloid PET: ^18^F-Flutemetamol uptake will be calculated using the Centiloid method.^88^ Additionally, scans will also be quantified regionally and categorised as either positive or negative according to visual reading following standard procedures.
- Tau (PET): Retention of ^18^F-RO6958948 in brain regions and voxel-based analysis of ^18^F-RO6958948 will be measured by in vivo PET. Thresholds for positivity will be applied and ^18^F-RO6958948 uptake will be categorised according to such values.

#### Clinical variables

- Neuropsychological testing: Cognition will be evaluated through a comprehensive neuropsychological battery, covering the main cognitive domains: (1) global cognition (MMSE, QI estimation (Word Accentuation Test)); (2) attention [Wechsler Adult Intelligence Scale (WAIS)-IV: Digit Span, Wechsler Memory Scale –Fourth Edition (WMS-IV): Symbol Span, Trail-Making Test (TMT)-A]; (3) episodic memory (Free and Cued Selective Reminding Test, Memory Binding Test, WMS-IV Logical Memory, NIH Toolbox Picture Sequence Memory Test); (4) executive function (TMT-B, Five Digits Test, WAIS-IV Coding, WAIS-IV Matrix Reasoning and NIH Toolbox Flanker Inhibition Test); (5) language (animal fluency: number of animals named in 1 min); and (7) visual processing (WAIS-IV Visual Puzzles, RBANS Judgement of Line Orientation).^89–94^
- Mood state: Mood state will be evaluated with the Spanish version of the Hospital Anxiety and Depression Scale.^95^
- Medical history, medication and lifestyle habits will be collected, including detailed neurological, psychiatric and systemic history; brief parental medical history; history of medication; and smoking, alcohol and drug consumption, physical exercise and dietary habits.
- Anthropometric measurements and vital signs will be acquired, including weight, height, waist diameter, body mass index (BMI), blood pressure and heart rate.
- Sex-specific variables including age at menopause, use of sexual hormones (oestrogens) to treat menopause symptoms and/or contraceptive medications, together with a history of surgeries and medication will be acquired.

### Statistical analyses

Data will be inspected to check normality assumptions and identify outliers. We will analyse associations between the following predictors: (1) objective (actigraphy, nasal flow monitoring device and PSG) and subjective sleep-related measures and (2) CSF–orexin-A levels; and the following dependent variables: (1) CSF AD and glial biomarkers, (2) neuroimaging and (3) cognitive performance outcomes. We will also analyse between-group differences in these predictors among different biomarker categories (AT profiles). These analyses will be adjusted by potential confounders (eg, age, sex, *APOE* genotype, education, anxiety/depression, BMI, sleep apnoeas and/or others) as appropriate. Additional interaction analyses may be performed to test whether *APOE* genotype or sex modify any of these associations. Mediation analyses will be used to evaluate whether associations between sleep-related metrics and brain structure and function are driven by AD and/or glial activity biomarkers. In addition, we will compute receiver operating characteristic curves and use the Youden Index (J=sensitivity+specificity–1) to derive optimal cut-off values for objective sleep measurements to discriminate between individuals with normal versus altered AD biomarkers. We will use general linear models to test associations involving continuous dependent variables and analysis of covariance (ANCOVA) to assess between-group differences among AT profiles. Non-parametric equivalents will be used if required model assumptions are not met.

#### Sample size calculation

**Figure 2:**
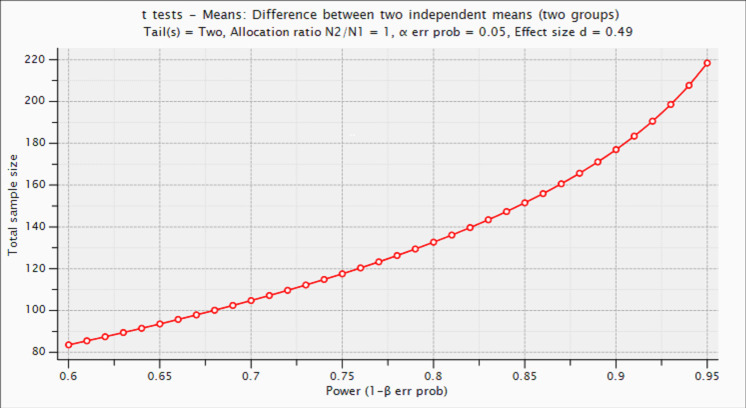
Estimation of statistical power as a function of sample size.

The sample size has been computed with G*Power V.3.1.9.4,^96^ taking into account the estimated effect size (ES) of a previous study using a similar methodology for the detection of differences in sleep efficiency between cognitively unimpaired individuals with normal versus abnormal Aβ biomarkers.^24^ For this ES (ES=0.49), we calculate that a sample size of n=178 (89 cases/89 controls) would be enough to achieve a statistical power of 90%, assuming a 5% type I error probability (two tails) (figure 2).

Based on these estimations, we plan to recruit 200 subjects: 100 with altered Aβ biomarkers and 100 with normal biomarkers. The sample size for the exploratory PSG substudy (n=90) is based on previous literature.^44^

### Patient and public involvement

Participants and the public were neither involved in the study design, recruitment or conduct, nor in the selection of research questions or study outcomes. All participants will receive feedback related to their usual sleep patterns. In case any clinically relevant finding is detected in the context of their participation, participants will receive a specific report describing such findings, as well as clinical counselling. Additionally, with the support of the communication unit at BBRC, we will develop specific activities to participate in public engagement initiatives. BBRC is fully aware of the importance of, and is committed to, involving the public as an active stakeholder of its research activities. As such, BBRC researchers lead and participate in diverse research-related communications and public awareness initiatives focused on increasing awareness about AD and the research that is being developed. BBRC will also include the profile of any proposed action at the institutional website, which is regularly updated as the research progresses.

### Ethics and dissemination

The ALFASleep study protocol has been approved by the independent Ethics Committee Parc de Salut Mar, Barcelona (2018/8207/I). All participants have signed a written informed consent before their inclusion (approved by the same independent ethics committee). Study findings will be presented at national and international conferences and submitted for publication in peer-reviewed journals.

## Discussion

Studies analysing the association between sleep and AD are gaining *momentum* since sleep represents both an appealing target for interventions aimed to prevent cognitive impairment and for the development of non-invasive diagnostic and prognostic biomarkers for AD. However, many studies published so far rely on subjective sleep quality scales, which are valuable clinical tools but may not be an ideal marker for incident dementia risk.^97^ Conversely, objective sleep measurements, such as actigraphy or PSG, provide unbiased, measurable and multidimensional sleep-quality data. Therefore, to gain a better understanding of the mechanisms underlying the association between sleep and cognitive impairment, there is a need for multimodal studies in cognitively unimpaired adults combining not only subjective but also objective sleep measures, with fluid biomarkers (encompassing AD and glial activation biomarkers, as well as CSF orexin levels), neuroimaging and cognitive data. Due to its multimodal approach and the profile of the study population (a cohort of cognitively unimpaired middle-aged adults enriched for AD risk), the ALFASleep project has the potential to make relevant contributions to the understanding of the role of sleep as a risk factor and early clinical manifestation of AD. On top of this, the present project may be complemented in the future with additional neuroimaging processing methods or MRI sequences to quantify structural changes in key regions involved in sleep–wake cycle regulation, such as the locus coeruleus or the hypothalamus,^98 99^ as well as emerging neuroimaging techniques to investigate the glymphatic system function, one of the most relevant potential links between sleep and AD pathophysiology.^20^ In addition, taking advantage of the prospective design of the ALFA+ study, with the present project, we intend to set the basis for a longitudinal study that may help unravel bidirectional associations between sleep and AD.

However, this project has some limitations. First, its observational design does not allow us to infer causality in potential associations between sleep and AD-related outcomes. Second, the ALFA+ cohort is mainly composed of well-educated individuals with very low comorbidity burden and low ethnic diversity, which may therefore constitute a source of bias.

Nonetheless, this project has direct potential clinical applications, which are the identification of altered sleep architecture patterns that may be specifically linked with AD pathology (such as decreased SWA), as well as characterising mechanisms linking sleep disruption and AD that may be amendable to potential therapeutic interventions. Thus, the resulting evidence could be used to design an intervention study targeting sleep quality, with the ultimate goal of preventing or slowing cognitive decline.

## Supplementary Material

Reviewer comments

## Data Availability

Data sharing is not applicable as no datasets were generated and/or analysed for this study. N/A.
