# Supplementary material for: Exploring cognitive and biological correlates of sleep quality and their potential links with Alzheimer’s disease (ALFASleep project): protocol for an observational study
Source: BMJ Open. 2022 Dec 30;12(12):e067159. doi: 10.1136/bmjopen-2022-067159 (PMC9809234; doi:10.1136/bmjopen-2022-067159)
Supplement: Supplementary data [file bmjopen-2022-067159supp001.pdf]

Supplementary Material

ALFA+ study exclusion criteria:

The ALFA+ protocol excludes those participants with any relevant medical condition associated with a life expectancy of less than 10 years and/or that may significantly interfere in the acquisition of data or interpretation of results. This includes:

1. Kidney failure on hemodialysis, liver cirrhosis, chronic lung disease with home oxygen therapy, solid organ transplant, fibromyalgia, active oncological disease under treatment (localized tumors are excluded.

2. Clinically relevant auditory and visual perception disorders that may interfere in cognitive assessments.

3. Major psychiatric disorders (according to the DSM-V manual) or diseases that affect cognitive abilities (major depressive episode, bipolar disorder, schizophrenia, and dementia).

4. Acquired brain damage: history of head trauma with macroscopic parenchymal or extra-axial injury, large-vessel ischemic or hemorrhagic stroke, brain tumors and other etiologies that can cause acquired brain damage (brain chemotherapy or radiotherapy).

5. Parkinson's disease, stroke, epilepsy under treatment and with frequent seizures (> 1/month) in the last year, multiple sclerosis or another neurodegenerative disease.

6. Contraindication for magnetic resonance imaging: claustrophobia, pacemaker carrier, history of ocular cerclage, carrier of any metal prosthesis incompatible with MRI (special care with heart valves).

7. Suspected family history of AD with autosomal dominant pattern (3 affected in two different generations) and early age of onset (<60 years). In this case, the participants will be referred to a specific genetic counseling program, since the family history suggests that it could be a case of monogenic AD.

8. Contraindication or technical impossibility of performing the LP at the time of inclusion in the study: patients undergoing treatment with oral anticoagulants, double antiplatelet therapy, coagulation disorders or thrombocytopenia, lumbar fixation, etc.

9. Radiological exclusion criteria.

10. Investigator criteria: participants who show any condition that, in the investigator's opinion, may interfere with the correct performance of the study procedures and/or future permanence in the study.

Actigraphy and nasal flow monitoring device data acquisition:

Participants will be instructed to wear an actigraph (Actiwatch2® Philips-Respironics, Murrysville, Pennsylvania) for two weeks in their non-dominant hand and press the event

marker button at bedtime and wake time. During this period, they will have to fulfill the sleep diary on a daily basis. They will be instructed to wear a nasal flow monitoring device (RUSleeping RTS®,, Philips-Respironics™, Murrysville, PA, USA) during the first night and annotate the results displayed in the device LCD screen on the next morning in the sleep diary. A study nurse will perform scheduled phone calls at days 1, 7 and 14 after baseline visit to ensure that data are being collected properly. After two weeks, the study materials will be sent back to BBRC facilities by courier service. The study personnel will download the data for further analyses.

Polysomnography acquisition:

PSG substudy participants will be instructed to keep to their usual sleep/wake rhythms and wear the actigraph one week before PSG acquisition (they will be also instructed to press the event marker button at bedtime and wake time). Participants will also be instructed to abstain from caffeine, alcohol, and daytime naps the previous 48h to the PSG acquisition.

Brain RT, OSG, Rumst, Belgium (http://www.osg.be/) will be used for PSG acquisition. The following parameters will be acquired:

- **EEG**: Right and left central (C3, C4), occipital (O1, O2) and frontal (F3, F4), referred to the contralateral ear mastoid side (M2, M1).
- **ECG**: single channel.
- **EOG**: Left and right electro-oculogram channels (E1-M1, E2-M1)
- **EMG**: surface electromyography of the mentalis muscle, bilateral anterior tibialis and bilateral flexor digitorum superficialis muscles.
- **Nasal and oral airflow**: Oronasal thermistor
- **Snore**: Microphone recording
- **Thoracic and abdominal movements**: Thoracic and abdominal effort belts
- **Continuous oxygen saturation**
- **Body position**

PSG recording parameters will be set as follows:

**Parameters**	**Sampling Rate**	**Impedance limit**
EEG	256 Hz	<5 KΩ
EOG	256 Hz	<5 KΩ
EMG	256 Hz	<10 KΩ
ECG	256 Hz	<10 KΩ
Airflow	32 Hz	-
Snore	256 Hz	-
Thoracic / abdominal effort belts	256 Hz	-
SpO2	4 Hz	-

The following step-by-step procedures will be performed since participants arrive until they leave the Sleep Unit:

- **Participant's preparation**: Participants will arrive at 20:30h to the Sleep Unit; EEG sensors will be placed and checked by technicians.
- **Dinner** will be offered at 20:45h, in the room. Participants will be allowed to relax, read and/or use their cell phone or similar devices until 22h.
- **Preparation of PSG recording:** At 22:00h, the remaining PSG components (EOG, EMG, ventilatory effort bands, pulsi oximeter, snore microphone) will be placed by technicians.
- **PSG calibration:** Participants will be instructed to lay supine and very still, with eyes open. Bio-calibrations will be performed prior to lights out and after lights on while recording. Data will be reviewed for artifacts, and electrodes/sensors will be changed as needed.
- **Wake EEG acquisition (wEEG):** During PSG calibration, wake EEG will be acquired. Participants will be first instructed to fix their gaze on a specific point that will be located always in the same position on the ceiling, so that they can look at it in a comfortable position. The following instructions will be given to prepare participants for wEEG acquisition*: "Now, we will record 3 min of data while you rest, there is no task to complete, just relax. You have to look at a fixed point in the ceiling. Find a comfortable position, stay calm, try to not move your head and relax the muscles on your face and your eyes."* When participant is ready, he/she will be asked *"Are you ready?"*. A quiet environment will be ensured, with technicians staying out from the visual field of the participant. A 3 min wEEG

recording block will start after the participant responds with OK. After, the participant will be asked to close eyes, and a second block of 3 minutes will start.

- **PSG recording:** Participants will be told they can go to sleep and lights will be turned off. Participants will be allowed to sleep until 7:30 AM. At this moment, lights will be turned on and they will be awakened (if still asleep), and PSG equipment will be removed.
- **PSG review:** PSG will be reviewed by a certified Sleep Medicine Neurologist and a report will be generated with sleep parameters and clinical findings. A clinical report with PSG results and any recommendations (if applicable) will be sent to each participant.

Sleep diary forms (questions are the same for the APP and paper version)

Form 1. Sleep habits (participants are instructed to fulfill this questionnaire every morning during two weeks)

After dinner and before bedtime, did you fall asleep on the couch, armchair, or other places other than your usual place to sleep?

○ Yes ○ No

What time did you go to bed with the intention of sleeping the night before?

hh:mm

How long did it take to fall asleep the night before?

Select... ▾ *Options: < = 15 min., 30 min., 60 min., 90 min., > = 120 min.*

At what time did you woke up this morning?

hh:mm

How many times did you woke up during the night?

Select ▾ *Options: None, 1, 2, 3, 4, 5, >5*

At what time did you got out of bed this morning?

hh:mm

Please, rate the quality of your sleep from 0 (worst possible quality) to 10 (best possible quality)

Select ▾ *Options: 0, 1, 2, 3, 4, 5, 6, 7, 8, 9, 10*

Did you nap or doze the day before?

○ Yes ○ No

Please, enter the approximate total time while you nap or doze the previous day (for example, if you took two 15-minute naps, mark 30 minutes) *(if applicable)*

Select ▾ *Options: 15 min., 30 min., 45 min., 60 min., 75 min., 90 min., ≥120 min.*

Did you take any prescribed or over-the-counter sleep medication the day before?

○ Yes ○ No

Please, indicate the name of the medicines you took *(if applicable)*

Free text

Please, indicate the total number of cigarettes, cigars, or pipes you consumed the day before

Select ▾ *Options: None, 1-5, 6-10, 10-15, 15-20, >20*

Please, indicate the total number of stimulant drinks you consumed the day before (coffee, tea, coke, energy drinks...)

Select ▾ *Options: None, 1, 2, 3, 4, 5, >5*

Did you drink any kind of alcoholic beverage the day before? *(if npt, applicable)*

○ Yes ○ No

Please, indicate the total number of beers (includes glasses, bottles or cans) *(if applicable)*

Select ▾ *Options: None, 1, 2, 3, 4, 5, 6, 7, 8, 9, 10, >10*

Please, indicate the total number of glasses of wine, champaigne, cider or "sangria" *(if applicable)*

Select ▾ *Options: None, 1, 2, 3, 4, 5, 6, 7, 8, 9, 10, >10*

Please, indicate the total number of glasses of aperitives (vermouth or similar), mixed drinks (gin and tonics...), brandy, liqueurs or whiskey *(if applicable)*

Select ▾ *Options: None, 1, 2, 3, 4, 5, 6, 7, 8, 9, 10, >10*

Please, indicate the total number of shots (liquor, whiskey...) *(if applicable)*

Select ▾ *Options: None, 1, 2, 3, 4, 5, 6, 7, 8, 9, 10, >10*

Comments

Free text

Form 2. RUSleeping RTS® scores* (participants are instructed to annotate all scores displayed in RUSleeping RTS® screen the morning after using this device)

Date of RUSleeping RTS data collection
dd/mm/yyyy

Does an error message display in the AH screen (AH - - -)?
○ Yes ○ No

Please, write down the number displayed in the AH screen
Numeric value

Does the HR1 screen display an error message (HR ERR 1 - - -)?
○ Yes ○ No

Please, write down the number that appears on the screen HR1
Numeric value

Does the HR2 screen display an error message (HR ERR 2 - - -)?
○ Yes ○ No

Please, write down the number that appears on the screen HR2
Numeric value

Does the HR3 screen display an error message (HR ERR 3 - - -)?
○ Yes ○ No

Please, write down the number that appears on the HR3 screen
Numeric value

Does the HR4 screen display an error message (HR ERR 4 - - -)?
○ Yes ○ No

Please, write down the number that appears on the HR4 screen
Numeric value

Does HR5 screen display an error message (HR ERR 5 - - -)?
○ Yes ○ No

Please, write down the number that appears on the screen HR5
Numeric value

Does the HR6 screen display an error message (HR ERR 6 - - -)?
○ Yes ○ No

Please, write down the number that appears on the screen HR6
Numeric value

Does the HR7 screen display an error message (HR ERR 7 - - -)?
○ Yes ○ No

Please, write down the number that appears on the HR7 screen
Numeric value

Does the HR8 screen display an error message (HR ERR 8 - - -)?
○ Yes ○ No

Please, write down the number that appears on the HR8 screen
Numeric value

Does the HR9 screen display an error message (HR ERR 9 - - -)?
○ Yes ○ No

Please, write down the number that appears on the HR9 screen
Numeric value

Comments
Free text

*This form is based on the RUSleeping RTS® "Response Card". This document, included in the RUSleeping RTS® User's Guide, is used to allow users to write their overnight results.

Form 3. Sleep-related additional questions for participant's roommate / bed partner*

1. Do you live with another person to whom you can ask about your sleep?

- ◯ Yes ◯ No

2. If you have a room mate or bed partner, please ask him/her how often during the last month have you experienced any of the following: *(if answer to the previous question is "No", the next questions are considered "not applicable")*

- **Loud snoring**
  - ◯ Not during the past month
  - ◯ Less than once a week
  - ◯ Once or twice a week
  - ◯ Three or more times a week
- **Large pauses between breaths while sleeping**
  - ◯ Not during the past month
  - ◯ Less than once a week
  - ◯ Once or twice a week
  - ◯ Three or more times a week
- **Jerking or twitching of the legs while sleeping**
  - ◯ Not during the past month
  - ◯ Less than once a week
  - ◯ Once or twice a week
  - ◯ Three or more times a week
- **Episodes of disorientation or confusion while sleeping**
  - ◯ Not during the past month
  - ◯ Less than once a week
  - ◯ Once or twice a week
  - ◯ Three or more times a week

*Adapted from Pittsburgh Sleep Quality Index (PSQI) questionnaire (item 10)
