# Supplementary material for: Exploring cognitive and biological correlates of sleep quality and their potential links with Alzheimer’s disease (ALFASleep project): protocol for an observational study
Source: BMJ Open. 2022 Dec 30;12(12):e067159. doi: 10.1136/bmjopen-2022-067159 (PMC9809234; doi:10.1136/bmjopen-2022-067159)
Supplement: Reviewer comments [file bmjopen-2022-067159.reviewer_comments.pdf]

PEER REVIEW HISTORY

BMJ Open publishes all reviews undertaken for accepted manuscripts. Reviewers are asked to complete a checklist review form (**http://bmjopen.bmj.com/site/about/resources/checklist.pdf**) and are provided with free text boxes to elaborate on their assessment. These free text comments are reproduced below.

ARTICLE DETAILS

**TITLE (PROVISIONAL)**	Exploring cognitive and biological correlates of sleep quality and their potential links with Alzheimer's disease (ALFASleep project): protocol for an observational study
**AUTHORS**	Fauria, Karine; Minguillón, Carolina; Knecevic, Iva; Tort-Colet, Núria; Stankeviciute, Laura; Hernández, Laura; Rădoi, Andreea; Deulofeu, Carme; Fuentes-Julián, Sherezade; Turull, Israel; Fusté, David; Sánchez-Benavides, Gonzalo; Arenaza-Urquijo, Eider; Suárez-Calvet, Marc; Holst, Sebastian; Garcés, Pilar; Mueggler, Thomas; Zetterberg, Henrik; Blennow, Kaj; Arqueros, Aurora; Iranzo, Álex; Domingo Gispert, Juan; Molinuevo, Jose; Grau-Rivera, Oriol

This article was not externally reviewed at BMJ Open. Protocols that have been independently assessed prior to submission to BMJ Open are usually fast-tracked to publication on the grounds that further substantial changes will not be possible. This independent assessment will usually be external, independent review for both a substantial grant award from a non-commercial or government funder and ethics approval.
